# Size-Dependent Transition from Stable Surface Modes to Symmetric Geometric Cleavage in Ultrasound-Driven Microbubbles

**DOI:** 10.3390/mi17030304

**Published:** 2026-02-28

**Authors:** Ruixiang Yu, Teng Zhang, Lianbin Zhao, Yongcheng Fang, Yongzhen Jin, Zihan Tang, Yumeng Feng, Yuanyuan Li, Hao Wu

**Affiliations:** 1 Innovative Institute of Chinese Medicine and Pharmacy, Shandong University of Traditional Chinese Medicine, Jinan 250355, China; yu_ruixiang@163.com (R.Y.); tengz_laz1112@163.com (T.Z.); zlb3657@163.com (L.Z.); jinyongzhen829@163.com (Y.J.); 2 School of Medical Informational Engineering, Shandong University of Traditional Chinese Medicine, Jinan 250355, China; fang_yongcheng@163.com (Y.F.); tangzihan_mail@163.com (Z.T.); fengyumeng23@163.com (Y.F.)

**Keywords:** acoustic cavitation, high-speed imaging, microfluidics, surface instabilities, symmetric fragmentation

## Abstract

The dynamic evolution of microbubbles under ultrasonic excitation is fundamental to applications ranging from targeted drug delivery to acoustic cleaning. This study employs a synchronous high-speed microscopic imaging system to systematically investigate the size-dependent stability and fragmentation of air microbubbles (*R*_0_ = 25–82.5 μm) in a free field at a driving frequency of 16.6 kHz. Our results demonstrate a clear mechanistic transition from stable radial oscillations to complex surface instabilities and, eventually, deterministic fragmentation. Smaller bubbles (*R*_0_ < 55 μm) exhibit long-term stability, transitioning through higher-order surface modes (*n* = 3 to *n* = 4) as surface energy accumulates. In contrast, larger bubbles (*R*_0_ > 60 μm) undergo violent non-spherical deformations characterized by centripetal necking and high-speed micro-jetting. Notably, we identify an inverse relationship between initial radius and fragmentation onset time, with larger bubbles reaching instability thresholds significantly earlier. Furthermore, a transition from stochastic breakup to bimodal, volume-symmetric splitting was observed as *R*_0_ increased, where daughter bubbles reached comparable volumes. These findings provide a theoretical and empirical basis for the controlled generation of monodisperse microbubble clouds, offering significant potential for enhancing the efficacy of ultrasonic contrast agents and therapeutic cavitation.

## 1. Introduction

Cavitation generally refers to the process in which pre-existing microscopic gas bubbles or nuclei in a liquid are activated under conditions of locally reduced pressure or external energy input, leading to rapid growth followed by violent oscillation and collapse. During the collapse stage, extremely high local pressures, transient high temperatures, strong shock waves, and microjets can be generated instantaneously [1]. Owing to these intense physical effects, cavitation has attracted considerable attention in a wide range of fields, including industrial processing, biomedical applications, and instrument design [2,3]. Cavitation can be initiated through various mechanisms, such as ultrasonic excitation, laser induction, fluid impact, and hydraulic low-pressure regions. Regardless of the trigger, single cavitation bubbles in an unbounded liquid exhibit universal dynamical behaviors and morphological evolution patterns [4,5].

Recent studies have demonstrated that temperature and ambient conditions play critical roles in governing the oscillation characteristics and collapse intensity of single cavitation bubbles. Experimental investigations combined with theoretical modeling under free-field conditions have revealed that increasing water temperature leads to larger maximum expansion radii and longer oscillation periods, while simultaneously weakening the collapse intensity. In extreme cases, bubble fragmentation into multiple microbubbles may occur, highlighting the significant influence of thermal effects on cavitation dynamics [6]. In addition, the dynamics of single cavitation bubbles vary markedly under different driving conditions. Numerical simulations have shown that, under ultrasonic excitation with sinusoidal, square, and triangular waveforms, the temporal evolution of bubble radius, the minimum collapse radius, and the corresponding peak internal pressure serve as key metrics for evaluating the dynamical response and energy distribution. These parameters are directly linked to the influence of driving waveforms on cavitation effectiveness [7]. In highly viscous media or complex fluids, microscopic bubble dynamics are further modified. Both numerical and experimental studies indicate that increasing liquid viscosity significantly suppresses the maximum bubble size, lifetime, and collapse dynamics [8]. Similar conclusions have been drawn from high-speed imaging experiments, which reveal distinct cavitation bubble evolution patterns and variations in collapse-induced shock wave intensity in liquids with different viscosities. These observations consistently demonstrate that liquid viscosity exerts a universal influence on bubble size and energy release behavior [9]. Collectively, these studies indicate that bubble size related parameters, including the initial radius, maximum expansion radius, and minimum collapse radius, constitute a unified framework for characterizing cavitation behavior across theoretical models, numerical simulations, and experimental observations. Importantly, these parameters not only provide quantitative measures of cavitation intensity but also serve as key variables linking environmental conditions, driving parameters, and the resulting cavitation effects [10]. Consequently, the stable generation and precise control of microbubbles have become central research topics in experimental cavitation studies in recent years.

With technological advancements, methods for generating cavitation microbubbles have evolved from traditional macroscopic hydrodynamic approaches toward more refined, controllable, and multiphysics-coupled strategies. Conventional macroscopic methods, such as gas sparging through porous plates or needle injection into bulk liquids, often suffer from high polydispersity and stochastic pinch-off dynamics due to turbulent fluctuations [11,12]. While recent developments in reciprocating syringe systems [13] and Venturi structures [14] have improved statistical characterization, they remain limited in precision compared to microscale fluidic control.

The emergence of microfluidic approaches—such as T-junctions [15,16], flow-focusing [17], and capillary-based geometries—has enabled highly controllable bubble formation by precisely regulating shear forces and interfacial tension. Unlike macroscopic systems, these micro-scale platforms operate within the quasi-static “dripping” regime, where the Capillary number (*Ca*) remains significantly below unity (*Ca* << 1). This ensures the production of highly monodisperse bubbles with coefficients of variation typically below 5% [18]. Such uniformity is a prerequisite for advanced applications like Contrast-Enhanced Ultrasound (CEUS) and targeted drug delivery, where the acoustic response is acutely sensitive to the initial bubble radius [19].

Among acoustically driven techniques, ultrasonic-induced bubble generation remains a core methodology [20]. However, decoupling the generation phase from the excitation phase is essential for isolating fundamental dynamics. The present study describes a novel experimental method utilizing a capillary-based microfluidic approach. By generating a micro-sized air bubble via a glass micropipette and syringe pump, we achieve precise control over the bubble’s initial size and spatial location. This method leverages the stability of microfluidic dripping to evaluate bubble dynamics under 16.6 kHz ultrasonic driving. Using high-speed photography (200,000–500,000 fps), we systematically investigate and compare the dynamics of air bubbles with different initial radii in a free field. This paper aims to: (1) propose a precision-controlled generation method for experimental cavitation research; and (2) characterize the *R*_0_-dependent transition of surface modes and fragmentation patterns in an acoustic field.

## 2. Experimental Setup

### 2.1. Experimental Configuration

**Figure 1:**
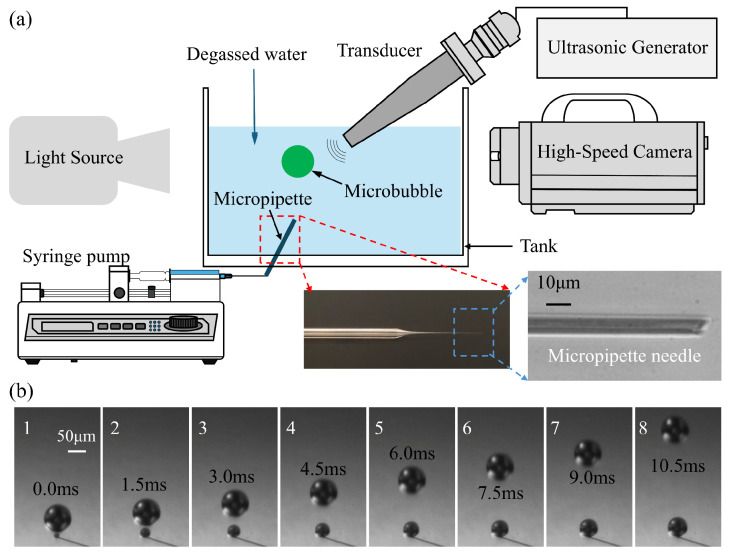
(**a**) The schematic description for experimental setup. (**b**) the process of bubbles’ generation near the micropipette connected to a syringe pump at a speed of 1 mL/min.

The schematic of the experimental system is illustrated in Figure 1a. A transparent, cuboid acrylic tank containing approximately 800 mL of degassed water was utilized, with the temperature maintained at 22 °C. The integrated setup, designated as the synchronous high-speed microscopic imaging system, comprises three primary modules: a high-speed camera system, a bubble generation apparatus, and ultrasonic devices. These components are centrally managed by a computer utilizing synchronous control techniques.

Experiments were performed using ultra-pure degassed water without the addition of surfactants. This choice was made to maintain the high surface tension (*σ* ≈ 72 mN/m) of the air-water interface, thereby isolating the intrinsic hydrodynamic instabilities and preventing the damping of surface modes by Marangoni stresses. The absence of surfactants ensures that the observed fragmentation thresholds and geometric cleavage patterns are representative of pure acoustic cavitation physics.

### 2.2. Optocal and Bubble Generation

The high-speed camera system consisted of a high-speed camera (Photron Inc., Tokyo, Japan; Fastcam SA-Z), lens (Cannon Inc., Tokyo, Japan; Macro Photo Lens, MP-E 65 mm) and light source (Edmund Optics, Barrington, NJ, USA; SugarCUBE™ LED Ultra). In the experiments of this study, the frame rate ranged from 200,000 to 500,000 fps to take into account both the details of the rapid movement of bubbles and the state of their long-term motion as the limitation of the camera’s storage. The spatial scale of the high-speed microscopic imaging system was calibrated such that each pixel represents a physical length of 5 μm. To account for potential non-spherical deformations during the initial rise phase, the equivalent diameter of each bubble was determined by averaging its orthogonal lengths measured along the horizontal and vertical axes. Consequently, the diameter measurement precision is ±5 μm, yielding a corresponding radial measurement uncertainty of ±2.5 μm.

Singe bubbles were generated with a glass micropipette connected to a syringe pump (KDS230, KD Scientific Incorporated, Holliston, MA, USA) through a syringe with a certain speed. The glass micropipette was pulled from a glass pipette to form an ultrafine tapered tip, as shown in the enlarged detail image in the bottom right corner of Figure 1a. The tip of the micropipette was put into the bottom of the tank and created a train of bubbles in the water. Figure 1b gives some photos of the bubbles generated with the micropipette when the syringe pump pulls the syringe at a speed of 1 mL/min. It can be seen that the bubble rises at a relatively low speed compared to the cavitation process. In present experiments, single bubbles were generated using a glass micropipette system coupled with a syringe pump. To evaluate the impact of initial bubble size on acoustic response, a parametric study was conducted by systematically varying the bubble radius (*R*_0_) from 25 to 82.5 μm. This was achieved through the controlled adjustment of the syringe pump flow rate (0.5 to 5.0 mL/min) and the selection of micropipettes with specific tip geometries. Under each set of parameters, the generation remained highly stable, ensuring that the microbubbles entering the acoustic field were monodisperse for each respective trial. An example of bubble generation with different initial radii under various pump flow rates is shown in Supplementary Figure S1. To characterize the stability of the bubble generation mode, the Capillary number (*Ca* = *μ*·*v*/*σ*) was evaluated, where *μ* is the dynamic viscosity of the water (≈10^−3^ Pa·s at 22 °C), *σ* is the surface tension of the water–air interface (≈72 × 10^–3^ N/m) and *v* is the characteristic velocity of the gas at the pipette tip. Given the fluid properties of degassed water and the characteristic velocities at the micropipette orifice (≈ 5 μm), *Ca* was found to be on the order of 10^−2^. This low *Ca* indicates a surface-tension-dominated “dripping” regime, which is mathematically associated with high monodispersity and reproducible pinch-off dynamics. This regime allowed for the precise parametric variation of *R*_0_ required for the subsequent acoustic fragmentation studies.

### 2.3. Acoustic Characterization and Synchronization

**Figure 2:**
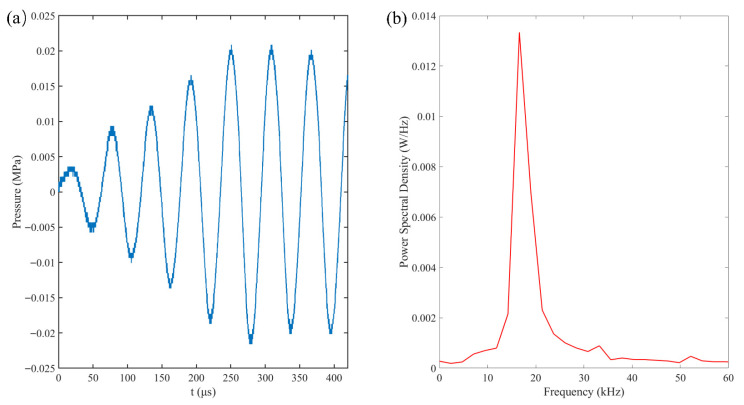
(**a**) Pressure–time profile that was recorded by the hydrophone near the rigid wall and (**b**) the corresponding frequency spectrum graph.

The acoustic field was generated by a custom-fabricated transducer with a resonant frequency of approximately 16.6 kHz, driven by a signal generator (RIGOL DG1022Z, Rigol Technologies Co. Ltd., Suzhou, China) and a power amplifier (PiezoDrive PD200 linear amplier, PiezoDrive, Shortland, NSW, Australia). Acoustic pressure was characterized by using a hydrophone (RESON TC4043, Teledyne RESON, Slangerup, Denmark) positioned at the bubble capture location (Figure 2a). As shown in the Fourier Transform results (Figure 2b), the pressure field is dominated by a single peak at 16.6 kHz. Pressure measurements indicated a brief transient phase, reaching a stable peak pressure of 0.022 MPa after approximately three cycles. To ensure the precise capture of the initial bubble evolution, a synchronization system utilizing a computer and a synchronizer was employed. This system coordinated the bubble generation, ultrasonic excitation, and high-speed imaging via Transistor-Transistor Logic (TTL) signals.

## 3. Results and Discussion

### 3.1. Experimental Observation of the Bubble Response in Free Field

**Figure 3:**
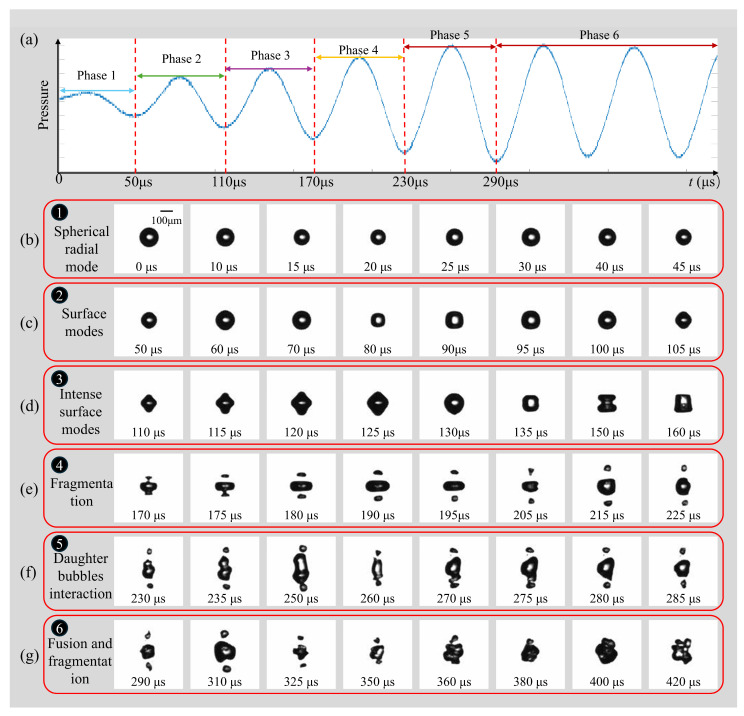
(**a**) The six phases are marked in the pressure–time profile. (**b**) Typical temporal evolution of a free-field individual bubble in phases of (**c**) oscillation; (**c**) surface modes; (**d**) intense surface modes; (**e**) fragmentation; (**f**) daughter bubble’s interaction; (**g**) fusion and fragmentation. (*R*_0_ = 72.5 μm, *f* = 16.6 kHz; *p_a_* = 0.022 MPa).

A representative response of an air microbubble (*R*_0_ = 72.5 μm) subjected to ultrasonic driving is illustrated in Figure 3 (see Supplementary Movie S1). Figure 3a depicts the ultrasonic pressure measured at the bubble’s equilibrium position. The acoustic frequency is *f* = 16.6 kHz, with a stable peak pressure of *p_a_* = 0.022 MPa; the wave propagation direction is from right to left in the images. Since the acoustic wavelength (*λ* ≈ 90 mm in water) is significantly larger than the bubble diameter (*λ*/2*R*_0_ ∼ *O*(10^2^)), the pressure field around the bubble is considered spatially uniform. In all figures, *t* = 0 denotes the onset of ultrasonic excitation. The temporal evolution of the bubble is shown in Figure 3b–g and can be categorized into six distinct phases. During the initial acoustic cycles (0–50 μs), the transducer operates in a transient state characterized by relatively low pressure. In this phase, the bubble oscillates exclusively in a stable, spherical radial mode (Figure 3b), maintaining its symmetry during compression and expansion. As the acoustic pressure increases during the second cycle (50–110 μs), the radial oscillation is accompanied by the onset of surface instabilities; specifically, mode *n* = 4 becomes discernible, where *n* represents the number of symmetric protrusions and indentations (Figure 3c). In the third cycle, as the driving pressure continues to rise, the bubble transitions fully into a high-amplitude surface mode (*n* = 4), while initially retaining a quasi-symmetrical shape (Figure 3d). However, this surface mode is inherently unstable. Under increasing pressure, the deformation amplitude grows rapidly, leading to the fragmentation of the bubble into three primary components during the fourth cycle (Figure 3e). Notably, due to gravitational effects, the upper boundary of the bubble exhibits higher surface velocities, resulting in the premature fragmentation of the top section. Post-fragmentation, the resulting daughter bubbles are sufficiently large to undergo mutual attraction via secondary Bjerknes forces within the same ultrasonic field. Because the generated fragments are typically in the sub-resonant regime relative to the 16.6 kHz driving frequency, they oscillate in phase. This phase-correlation results in a mutual attraction that drives the convergence of daughter bubbles toward the central ‘main’ bubble, eventually culminating in the formation of a localized cavitation cloud. At the onset of this interaction, the three bubbles oscillate in distinct surface modes at their respective positions with no immediate coalescence (Figure 3f). As their motion intensifies, the peripheral bubbles migrate toward the center and merge with the primary bubble. Throughout this fusion process, continuous secondary fragmentation occurs, eventually culminating in the formation of a localized bubble cloud (Figure 3g). While these observations pertain to a 72.5 μm bubble at low driving amplitudes, bubbles of varying sizes typically undergo a similar sequence, experiencing all or a subset of these six evolutionary phases.

### 3.2. Stable Surface Modes of Smaller Bubbles in Free Field

**Figure 4:**
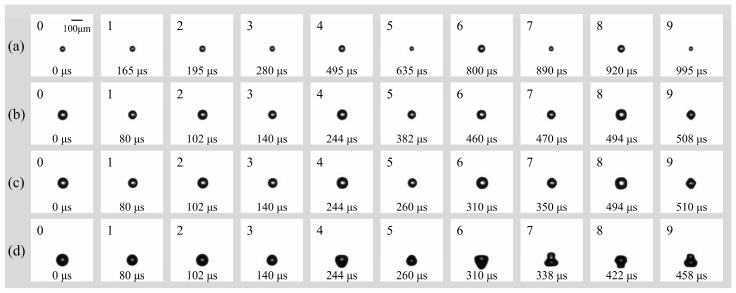
(**a**) Temporal evolution of individual bubbles with (**a**) *R*_0_ = 25 μm; (**b**) *R*_0_ = 40 μm; (**c**) *R*_0_ = 47.5 μm; (**d**) *R*_0_ = 52.5 μm in free field under the irradiation of ultrasound (*f* = 16.6 kHz, *p_a_* = 0.022 Mpa).

Previous studies have established that surface modes remain stable at smaller bubble radii and lower driving amplitudes, whereas increases in either parameter tend to induce non-spherical instabilities [21]. In the present study, the peak ultrasonic pressure was maintained at *p_a_* = 0.022 MPa (*f* = 16.6 kHz) to investigate the surface modes of smaller microbubbles in a free field. Figure 4 illustrates the dynamics of bubbles with initial radii (*R*_0_) ranging from 25 to 52.5 μm. For a bubble with *R*_0_ = 25 μm (Figure 4a), stable surface modes were observed for over 1 ms (the observation window was limited to 1.2 ms by the camera’s storage capacity at 200 kfps). During the initial 14 acoustic cycles (Figure 4a: 1–6), the bubble underwent continuous radial contraction and expansion at a fixed position while maintaining a spherical geometry. As the bubble continued to pulsate, the accumulation of energy eventually triggered a transition into higher-order surface modes. Specifically, the mode parameter *n* evolved from *n* = 3 (Figure 4a: 7–8) to *n* = 4 (Figure 4a: 9). The dynamics for bubbles with *R*_0_ = 40 μm and 47.5 μm (Figure 4b and Figure 4c, respectively) were qualitatively similar to those described above. The primary distinction was the temporal onset of the surface modes: the transition to *n* = 4 occurred earlier as the initial radius increased, appearing at 470 μs for *R*_0_ = 40 μm and at 350 μs for *R*_0_ = 47.5 μm. In the case of *R*_0_ = 52.5 μm (Figure 4d), the bubble sequentially transitioned from a purely radial oscillation phase (Figure 4d: 0–2) to a surface mode of *n* = 3 (Figure 4d: 3–6). During the observation period, the bubble entered a more vigorous oscillation state, transitioning from a prolate, droplet-like geometry to a highly deformed, multi-lobed (gourd-like) configuration (Figure 4d: 7–9). Notably, for this larger radius, the surface mode emerged significantly earlier (approximately 140 μm), exhibiting higher-amplitude surface deformations compared to the smaller bubbles.

### 3.3. Fragmentation Characteristics of Bigger Bubbles in a Free Field

As the bubble size continues to increase, during the camera observation period (525 μs), bubble motion will exhibit bubble fragmentation phenomena following the spherical radial mode and surface mode. The daughter bubbles, due to their different sizes, will exhibit varying motion behaviors. The smaller bubbles, with limited attraction from the parent bubble, will gradually rise and move away from the main bubble. In contrast, the larger bubbles will undergo symmetric motion around the main bubble and influenced by the main bubble’s movement, will gradually merge back with it. The main bubble is defined as the bubble that is closest to the position of the original (undivided) bubble after fragmentation, while the bubbles resulting from the fragmentation are defined as daughter bubbles. In this section, we will analyze in detail the unstable motion characteristics of ultrasound cavitation microbubbles in a free field as their radius increases. This includes bubble fragmentation and the subsequent motion of the resulting smaller bubbles. The study in this part is crucial for understanding the evolution process of ultrasound cavitation microbubbles from large bubbles to tiny bubble clouds, and it provides important guidance for the generation and further application of micro- and nanobubbles.

**Figure 5:**
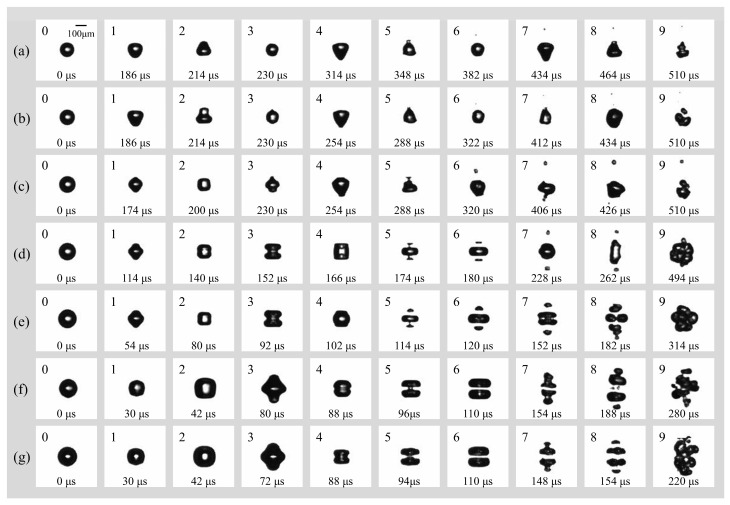
(**a**) Temporal evolution of an individual bubble with (**a**) *R*_0_ = 60 μm; (**b**) *R*_0_ = 65 μm; (**c**) *R*_0_ = 70 μm; (**d**) *R*_0_ = 72.5 μm; (**e**) *R*_0_ = 77.5 μm; (**f**) *R*_0_ = 80 μm; (**g**) *R*_0_ = 82.5 μm in free field under the irradiation of ultrasound (*f* = 16.6 kHz, *p_a_* = 0.022 Mpa).

Figure 5 illustrates the morphological evolution of cavitation bubbles in a free field under a peak acoustic pressure of 0.022 MPa and an ultrasonic frequency of 16.6 kHz. The initial radii *R*_0_ increase from Figure 5a–g, ranging from 60 μm to 82.5 μm. It is obvious that the transition from spherical symmetry (spherical radial mode) to complex non-spherical oscillation (surface mode) is highly dependent on the initial radius *R*_0_. For bubbles in small radius regime range (Figure 5a–c, *R*_0_ = 60 μm, 65 μm, and 70 μm), they maintain relatively high spherical stability during the initial spherical radial mode. Surface instabilities emerge primarily during the later surface mode phase, characterized by low-order modes (likely *n* = 2 or *n* = 3). This results in “pear-shaped” or slightly triangular deformations. For bubbles in larger radius regime range (Figure 5d–g, *R*_0_ = 72.5 μm, 77.5 μm, 80 μm, and 82.5 μm), they exhibit violent nonlinear oscillations as *R*_0_ increases and have a higher surface mode order (*n* = 4). The surface rapidly evolves into multi-polar distributions. In Figure 5f,g, the bubbles adopt “disk-like” or “flattened” geometries early in the cycle, indicating that larger bubbles are more susceptible to high-order surface wave excitations under these acoustic conditions.

**Figure 6:**
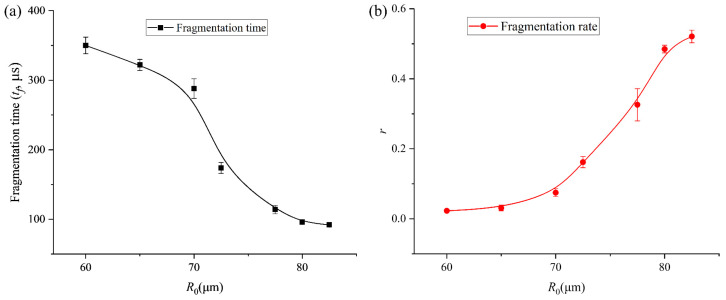
(**a**) Results of fragmentation time of bubble with different initial radii. (**b**) Results of the volume ratio (*r*) of the daughter bubbles and the main bubble after the first fragmentation of the bubble with various initial radii.

In addition, the fragmentation mechanism undergoes a distinct transition from stochastic rupture to organized geometric cleavage as *R*_0_ increases. For smaller bubbles (Figure 5a–c, *R*_0_ = 60 μm, 65 μm, and 70 μm), the fragmentation process is often driven by a single micro-jet or localized surface instability, leading to a disorganized break-up. For bubbles with *R*_0_ = 72.5 μm, 77.5 μm, 80 μm, and 82.5 μm (Figure 5d–g), a consistent centripetal necking effect is observed. The bubble elongates and narrows at its center, forming a “dumbbell” shape before snapping into two or more primary fragments. Interestingly, there is a notable inverse relationship between *R*_0_ and the fragmentation time which has been analyzed and shown in Figure 6. The bubble in Figure 5g reaches total collapse/fragmentation by 154 μs, significantly faster than the 382 μs observed in Figure 5g, suggesting that larger bubbles reach their resonance or instability threshold much earlier in the pressure cycle.

After fragmentation, the post-fragmentation multi-bubble dynamics are also interesting and different as *R*_0_ increases. The behavior of the resulting daughter bubbles reveals a transition into complex cavitation clouds: firstly, larger initial bubbles produce a higher number of daughter bubbles that exhibit strong spatial correlation. In Figure 5f,g, the fragments appear as organized “bead-like” strings or layered clusters, reflecting the underlying symmetry of the parent bubble’s collapse; secondly, though all cases eventually evolve into a bubble cloud (frames 9 of Figure 5a–g), the clouds originating from larger bubbles are denser and more widespread, representing a more efficient conversion of acoustic energy into surface area and localized mechanical energy. Under the influence of Laplace pressure (*P_L_* = 2*σ*/*R*_0_), the microbubble would be inherently metastable, as the elevated internal pressure facilitates the diffusion of gas into the surrounding liquid. Over an infinite duration, this process leads to total dissolution. However, the “stability” reported in this study refers to a dynamic equilibrium maintained by the 16.6 kHz ultrasonic field. In this state, the energy input from the acoustic field sustains the bubble population through rectified diffusion and prevents total dissolution, while the interplay of fragmentation and Bjerknes-force-induced coalescence establishes a temporary steady-state size distribution. The system thus resides in a non-equilibrium steady state (NESS) as long as enough external energy input persists or the bubble keeps in the ultrasonic field.

To further characterize the relationship between bubble fragmentation characteristics and the initial bubble radius, we continued to collect data on the time at which bubbles of different initial sizes begin to split, as well as the relationship between the volume ratio (*r*) of the daughter bubbles and the main bubble when both the daughter bubbles and the main bubble firstly reach their maximum expansion after fragmentation, as shown in Figure 6a and b, respectively. As the initial bubble size increases, the onset time of bubble fragmentation under ultrasound gradually occurs earlier. Meanwhile, after fragmentation, the size difference between the daughter bubble and the main bubble progressively decreases—from an initial volume disparity of nearly two orders of magnitude to comparable volumes—and eventually the bubble undergoes volume symmetric splitting, producing two bubbles of nearly identical size. The experimental trends observed in Figure 6 can be characterized by the competition between the disruptive acoustic pressure and the stabilizing interfacial tension. The restorative capacity of the bubble is represented by its Laplace pressure, *P_L_* = 2σ/*R*. As *R*_0_ increases from 25 μm to 82.5 μm, *P_L_* decreases, rendering the interface more susceptible to high-order modal excitations (*n* = 4). The decrease in fragmentation time (Figure 6a) for larger bubbles is a direct result of this reduced stabilization; the acoustic energy overcomes the surface energy barrier earlier in the expansion–compression cycle. Furthermore, the convergence of the volume ratio *r* toward unity (Figure 6b) indicates a transition in the dominant fragmentation mode. In the large *R*_0_ regime, the fragmentation is governed by a global geometric instability (centripetal necking) that minimizes the energy required to create new surface area by splitting the volume into two nearly equal spheres. In contrast, for smaller *R*_0_, the high surface tension restricts fragmentation to localized, high-curvature regions, leading to the ejection of asymmetric daughter bubbles.

The transition from stable radial oscillation to geometric cleavage is a result of parametric instability. The growth of surface distortions *a_n_* is driven by the acceleration of the bubble interface $\ddot{R}$. For our 16.6 kHz system, the fragmentation threshold is reached when the surface tension energy barrier is surpassed by the acoustic work. As the initial radius *R*_0_ increases, the Laplace pressure 2*σ*/*R*_0_ decreases linearly. This reduction in the restoring force allows for a faster accumulation of energy into the *n* = 4 mode, which we observe as a decrease in fragmentation time as a function of *R*_0_ (Figure 6a).

These findings provide important guidance for further promoting the symmetric fragmentation of microbubbles to generate size-uniform microbubbles for practical applications, particularly in clinical medicine. In addition, we hypothesize that the initial bubble radius required to induce volume-symmetric fragmentation may be related to the ultrasound frequency, which constitutes an important direction for our future research.

## 4. Conclusions

This study systematically investigated the dynamic response of air microbubbles in a free field under ultrasonic driving (*f* = 16.6 kHz, *p_a_* = 0.022 MPa). Our observations reveal a clear, size-dependent transition in bubble behavior, categorized into six distinct regimes: stable radial oscillation, stable surface modes, high-order surface mode evolution, fragmentation, daughter bubbles interaction, fusion and fragmentation.

For smaller bubbles (*R*_0_ < 52.5 μm), surface instabilities remain energetically constrained, resulting in a gradual transition between modes *n* = 3 to *n* = 4 without interfacial rupture. As the initial radius increases (*R*_0_ > 60 μm), the bubble dynamics transition into a highly nonlinear and violent regime. We identified a critical shift in the fragmentation mechanism: smaller cavitation bubbles undergo stochastic, jet-driven breakup, while larger bubbles exhibit a highly organized centripetal necking process. This “dumbbell-shaped” cleavage leads to a significant reduction in the fragmentation onset time as a function of *R*_0_.

Most notably, our results demonstrate that increasing the initial bubble size promotes a transition toward volume-symmetric splitting. At the upper end of the investigated radius range (*R*_0_ ≈ 82.5 μm), the volume disparity between daughter bubbles and the main bubble vanishes, yielding fragments of nearly identical size. This discovery offers a strategic framework for the controlled generation of monodisperse microbubbles, which is of paramount importance for therapeutic and diagnostic applications in clinical medicine. Future research will explore the coupling effects between ultrasonic frequency and the critical radius to further refine the predictability of these fragmentation patterns.

## Data Availability

The original contributions presented in this study are included in the article/Supplementary Material. Further inquiries can be directed to the corresponding authors.

## Supplementary Materials

The following supporting information can be downloaded at: https://www.mdpi.com/article/10.3390/mi17030304/s1, Figure S1: Bubble generation with different pump flow rate: (a) 1 mL/min; (b) 2 mL/min; (c) 3 mL/min; Movie S1: Single bubble dynamics in free field under the irritation of low-frequency ultrasound (*R*_0_ = 72.5 μm).
