# Supplementary figures and images for: Size-Dependent Transition from Stable Surface Modes to Symmetric Geometric Cleavage in Ultrasound-Driven Microbubbles
Source: Micromachines (Basel). 2026 Feb 28;17(3):304. doi: 10.3390/mi17030304 (PMC13028647; doi:10.3390/mi17030304)

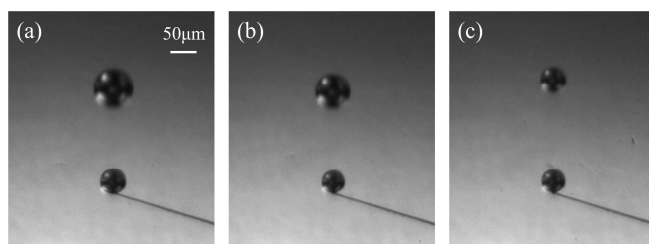

Figure S1. Bubble generation with different pump flow rate: (a) 1 ml/min; (b) 2 ml/min; (c) 3 ml/min.

Supplement: Supplementary file 1 [file micromachines-17-00304-s001.zip › Supplementary Figure S1.pdf]
